# Phosphodiesterase type 4 inhibition enhances nitric oxide- and hydrogen sulfide-mediated bladder neck inhibitory neurotransmission

**DOI:** 10.1038/s41598-018-22934-1

**Published:** 2018-03-16

**Authors:** Ángel Agis-Torres, Paz Recio, María Elvira López-Oliva, María Pilar Martínez, María Victoria Barahona, Sara Benedito, Salvador Bustamante, Miguel Ángel Jiménez-Cidre, Albino García-Sacristán, Dolores Prieto, Vítor S. Fernandes, Medardo Hernández

**Affiliations:** 1 Departamento de Fisiología, Facultad de Farmacia, Universidad Complutense de Madrid, 28040 Madrid, Spain; 2 Departamento de Anatomía y Anatomía Patológica Comparadas, Facultad de Veterinaria, Universidad Complutense de Madrid, 28040 Madrid, Spain; 3 Departamento de Toxicología y Farmacología, Facultad de Veterinaria, Universidad Complutense de Madrid, 28040 Madrid, Spain; 4 Servicio de Urología, Hospital Universitario Puerta de Hierro-Majadahonda, 28222 Madrid, Spain; 5 Servicio de Urología, Hospital Universitario Ramón y Cajal, 28034 Madrid, Spain

## Abstract

Nitric oxide (NO) and hydrogen sulfide (H_2_S) play a pivotal role in nerve-mediated relaxation of the bladder outflow region. In the bladder neck, a marked phosphodiesterase type 4 (PDE4) expression has also been described and PDE4 inhibitors, as rolipram, produce smooth muscle relaxation. This study investigates the role of PDE4 isoenzyme in bladder neck gaseous inhibitory neurotransmission. We used Western blot and double immunohistochemical staining for the detection of NPP4 (PDE4) and PDE4A and organ baths for isometric force recording to roflumilast and tadalafil, PDE4 and PDE5, respectively, inhibitors in pig and human samples. Endogenous H_2_S production measurement and electrical field stimulation (EFS) were also performed. A rich PDE4 and PDE4A expression was observed mainly limited to nerve fibers of the smooth muscle layer of both species. Moreover, roflumilast produced a much more potent smooth muscle relaxation than that induced by tadalafil. In porcine samples, H_2_S generation was diminished by H_2_S and NO synthase inhibition and augmented by roflumilast. Relaxations elicited by EFS were potentiated by roflumilast. These results suggest that PDE4, mainly PDE4A, is mostly located within nerve fibers of the pig and human bladder neck, where roflumilast produces a powerful smooth muscle relaxation. In pig, the fact that roflumilast increases endogenous H_2_S production and EFS-induced relaxations suggests a modulation of PDE4 on NO- and H_2_S-mediated inhibitory neurotransmission.

## Introduction

Phosphodiesterases (PDEs) are enzymes that hydrolyze cyclic nucleotides (cAMP and cGMP), so that PDE inhibition causes augment in intracellular [cAMP] and/or [cGMP] and consequent kinase activation, thus causing smooth muscle relaxation^1,2^. 11 types of PDEs (PDE1-PDE11) have been described, of which the PDE4 isoenzyme specifically hydrolyze cAMP and PDE5 is cGMP-selective PDE^1,2^.

The cAMP pathway is crucial in the control of urinary bladder smooth muscle tension. cAMP, via PKA activation, regulates smooth muscle function by targeting the activity of several K^+^ and Ca^2+^ channels, thus decreasing the smooth muscle excitability and contractility^2–8^. While the cAMP pathway seems to play a key role in detrusor relaxation so that PDE4 inhibitors have been suggested for the treatment of bladder overactivity, the NO/cGMP signaling would be involved in regulating urethral contractility. In fact PDE5 inhibitors, such as tadalafil, are useful for the treatment of lower urinary tract symptoms (LUTS) associated with benign prostatic enlargement (LUTS/BPE)^9–19^.

In the bladder outflow region, nitric oxide (NO)^20–22^ and hydrogen sulfide (H_2_S)^23,24^ play a pivotal role in nerve-mediated relaxation. Along with these mediators, other non-gaseous molecules, such as adenosine 5′-triphosphate (ATP)^25^, serotonin^26^ and peptides, such as pituitary adenylate cyclase-activating polypeptide 38^27,28^, are also implicated in the bladder neck smooth muscle relaxation. Our lab has previously described a marked PDE4 expression in pig and human bladder outflow region, where the selective PDE4 inhibitor rolipram causes a powerful smooth muscle relaxation^29^. The study of the underlying mechanisms regulating the bladder neck smooth muscle tension is necessary to provide drugs causing bladder outlet region relaxation during the voiding in obstructive LUTS^20^.

Roflumilast is an orally active PDE4 inhibitor with anti-inflammatory and bronchodilator effects approved for the treatment of severe chronic obstructive pulmonary disease (COPD)^30–33^. As the PKA signaling pathway might represent a valuable therapeutic target for patients suffering LUTS/BPE, the present study was designed to investigate the role played by PDE4 isoenzyme and PDE4 inhibitors, such as roflumilast, in bladder neck gaseous inhibitory neurotransmission.

## Results

### Expression of neuronal NPP4 (PDE4) and PDE4A

**Figure 1:**
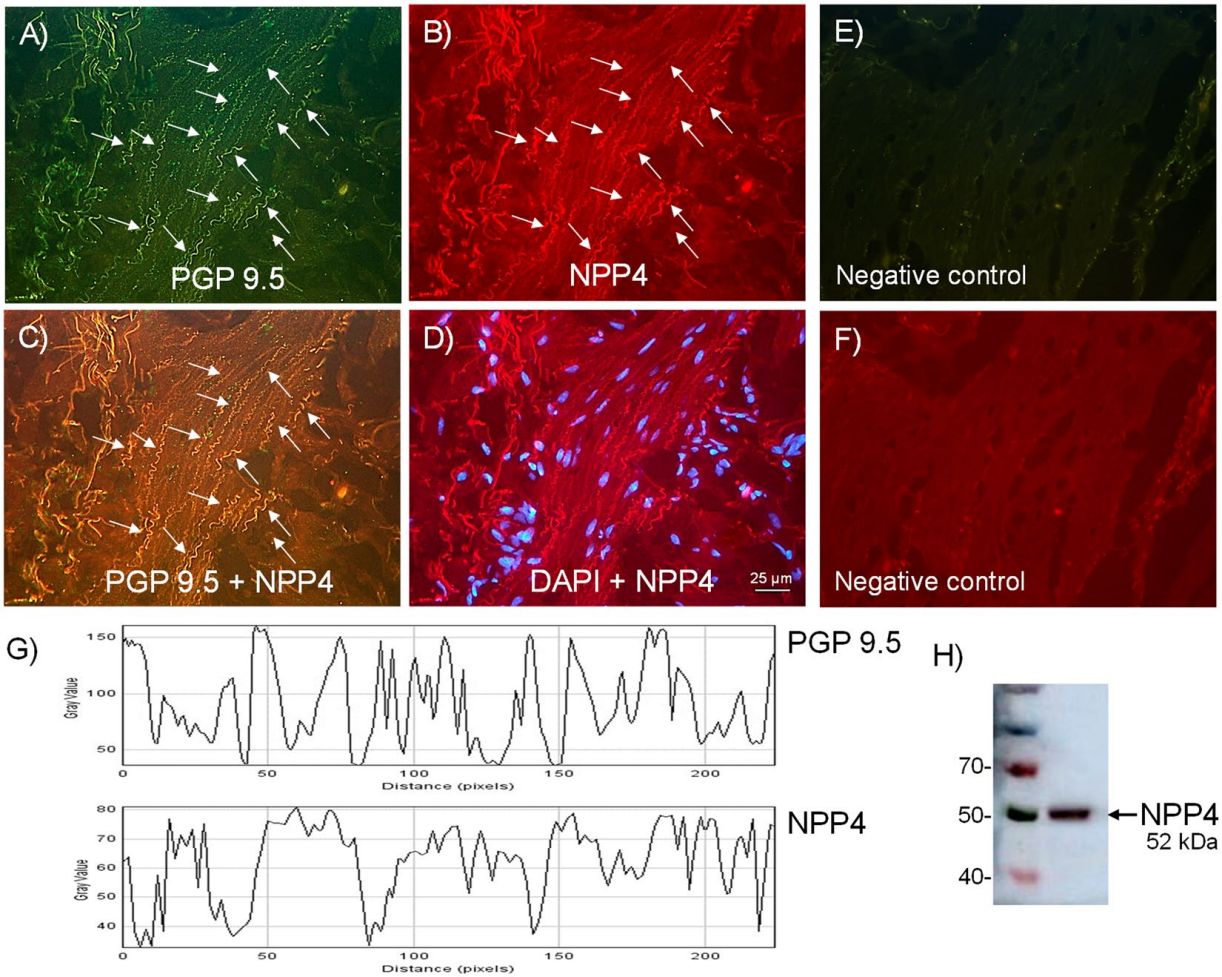
Expression of PDE4 (NPP4) protein within nerve fibers distributed among pig bladder neck smooth muscle bundles. Double-labeling immunofluorescence assay in the pig bladder neck (**A**–**D**). Bladder neck overall innervation was visualized using the general nerve marker PGP 9.5 (green areas) (**A**). NPP4 immunofluorescence from pig bladder neck reveals immunopositive nerve trunks (red areas), running parallel to the smooth muscle bundles. Same fields (**A**,**B**,**E** and **F**). Immunofluorescence double-labeling for PGP 9.5 and NPP4 in the smooth muscle, demonstrate neuronal co-localization (yellow areas) (**C**). Cell nuclei were counterstained with DAPI (blue areas) (**D**). Scale bars indicate 25 µm. Negative controls showing the lack of immunoreactivity in sections incubated in the absence of the primary antibody (**E** and **F**) (n = 5). Comparison of the fluorescence of PGP 9.5 and NPP4, using ImageJ, which shows a predominant co-localization between NPP4 and PGP 9.5 (**G**). Western blot of smooth muscle membranes from pig bladder neck incubated with NPP4 antibody showing a 52 kDa major band, which corresponded to the expected molecular weight (**H**) (n = 5).

**Figure 2:**
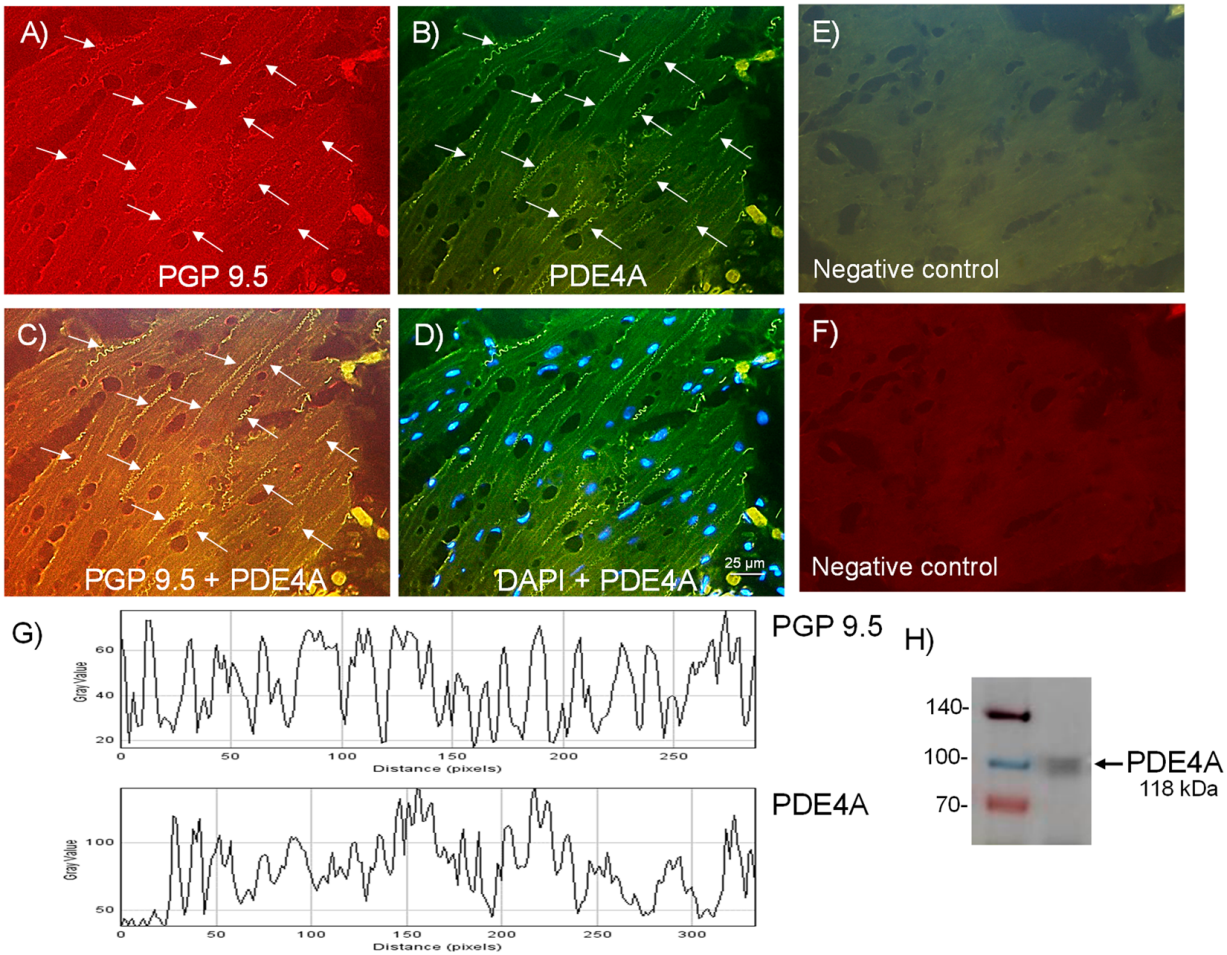
Expression of PDE4A protein within nerve fibers distributed among pig bladder neck smooth muscle bundles. Double-labeling immunofluorescence assay in the pig bladder neck (**A**–**D**). Bladder neck overall innervation was visualized using the general nerve marker PGP 9.5 (red areas) (**A**). PDE4A immunofluorescence from pig bladder neck reveals immunopositive nerve trunks (green areas), running parallel to the smooth muscle bundles. Same fields (**A**,**B**,**E** and **F**). Immunofluorescence double-labeling for PGP 9.5 and PDE4A in the smooth muscle, demonstrate neuronal co-localization (yellow areas) (**C**). Cell nuclei were counterstained with DAPI (blue areas) (**D**). Scale bars indicate 25 µm. Negative controls showing the lack of immunoreactivity in sections incubated in the absence of the primary antibody (**E** and **F**) (n = 5). Comparison of the fluorescence of PGP 9.5 and PDE4A, using ImageJ, which shows a major co-localization between PDE4A and PGP 9.5 (**G**). Western blot of smooth muscle membranes from pig bladder neck incubated with PDE4A antibody showing a 118 kDa major band, which corresponded to the expected molecular weight (**H**) (n = 5).

**Figure 3:**
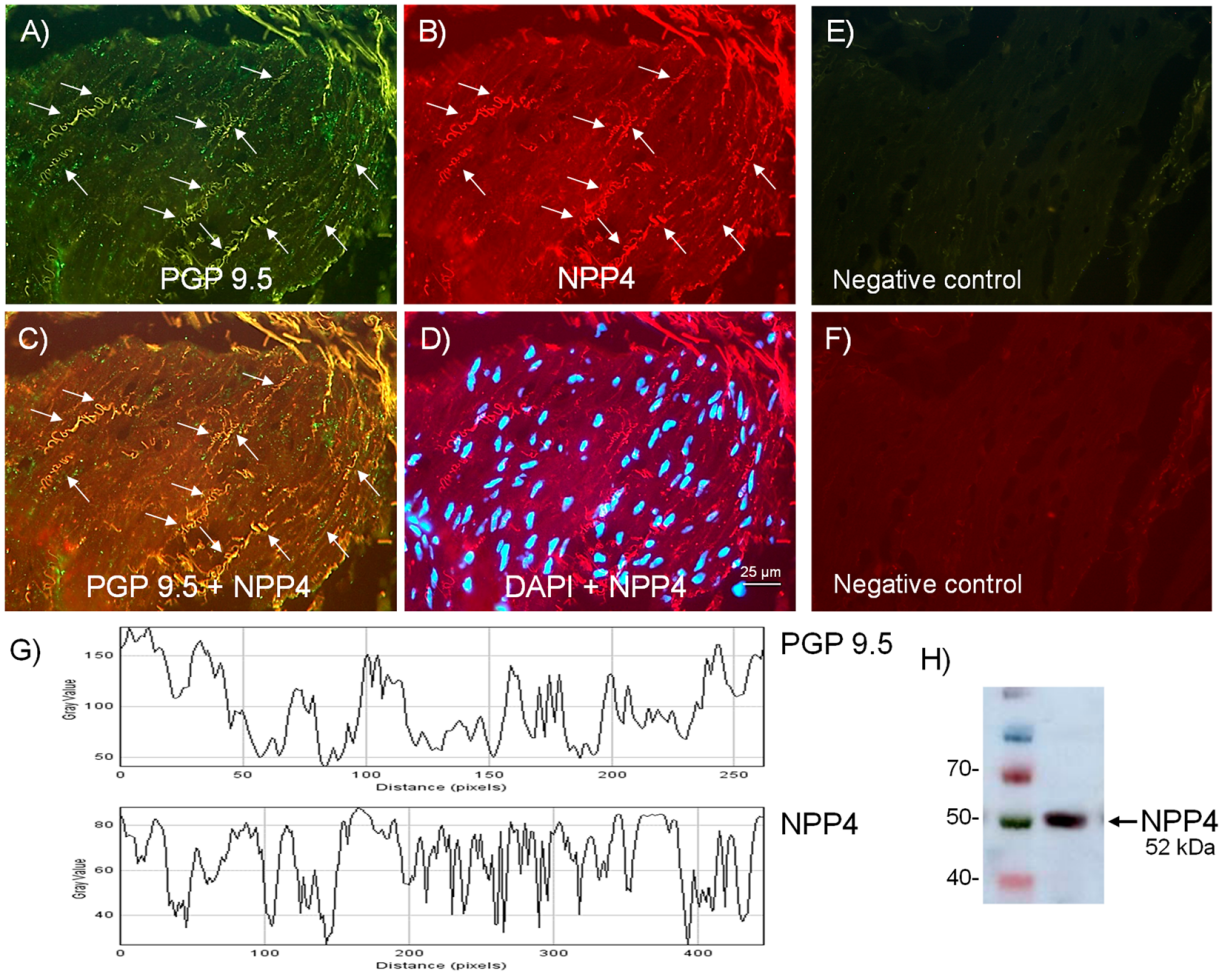
Expression of PDE4 (NPP4) protein within nerve fibers distributed among human bladder neck smooth muscle bundles. Double-labeling immunofluorescence assay in the human bladder neck (**A**–**D**). Bladder neck overall innervation was visualized using the general nerve marker PGP 9.5 (green areas) (**A**). NPP4 immunofluorescence from human bladder neck reveals immunopositive nerve trunks (red areas), running parallel to the smooth muscle bundles. Same fields (**A**,**B**,**E** and **F**). Immunofluorescence double-labeling for PGP 9.5 and NPP4 in the smooth muscle, demonstrate neuronal co-localization (yellow areas) (**C**). Cell nuclei were counterstained with DAPI (blue areas) (**D**). Scale bars indicate 25 µm. Negative controls showing the lack of immunoreactivity in sections incubated in the absence of the primary antibody (**E** and **F**) (n = 4). Comparison of the fluorescence of PGP 9.5 and NPP4, using ImageJ, which shows a priority co-localization between NPP4 and PGP 9.5 (**G**). Western blot of smooth muscle membranes from human bladder neck incubated with NPP4 antibody showing a 52 kDa major band, which corresponded to the expected molecular weight (**H**) (n = 4).

**Figure 4:**
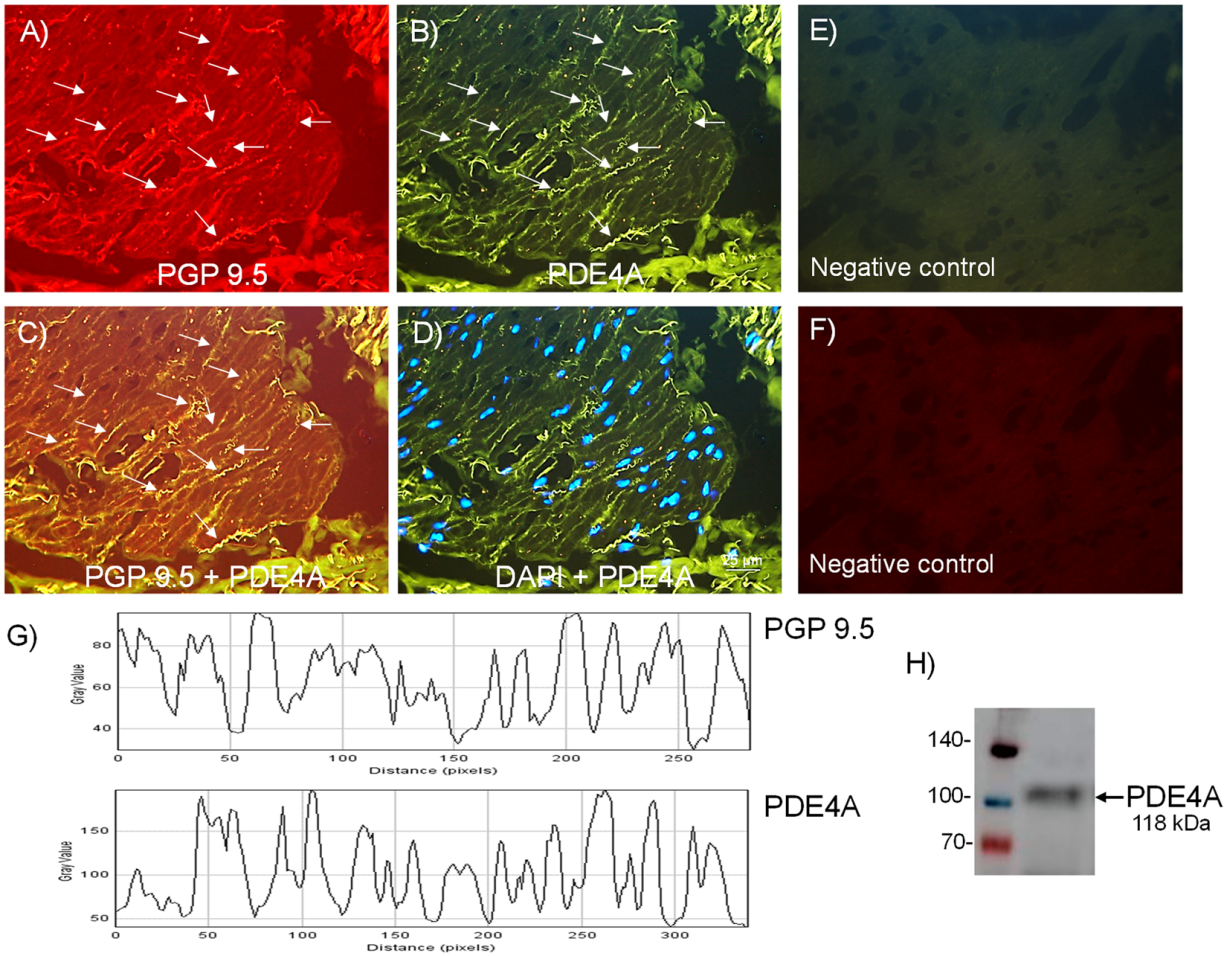
Expression of PDE4A protein within nerve fibers distributed among human bladder neck smooth muscle bundles. Double-labeling immunofluorescence assay in the human bladder neck (**A**–**D**). Bladder neck overall innervation was visualized using the general A marker PGP 9.5 (red areas) (**A**). PDE4A immunofluorescence from human bladder neck reveals immunopositive nerve trunks (green areas), running parallel to the smooth muscle bundles. Same fields (**A**,**B**,**E** and **F**). Immunofluorescence double-labeling for PGP 9.5 and PDE4A in the smooth muscle, demonstrate neuronal co-localization (yellow areas) (**C**). Cell nuclei were counterstained with DAPI (blue areas) (**D**). Scale bars indicate 25 µm. Negative controls showing the lack of immunoreactivity in sections incubated in the absence of the primary antibody (**E** and **F**) (n = 4). Comparison of the fluorescence of PGP 9.5 and PDE4A, using ImageJ, which shows a major co-localization between PDE4A and PGP 9.5 (**G**). Western blot of smooth muscle membranes from human bladder neck incubated with PDE4A antibody showing a 118 kDa major band, which corresponded to the expected molecular weight (**H**) (n = 4).

Neuronal NPP4 (PDE4) and PDE4A expression in pig (n = 5 pigs) and human (n = 4 persons) bladder neck samples were investigated by double staining immunohistochemistry using NPP4 and PDE4A selective antibodies together with pan-neuronal marker protein gene product (PGP) 9.5. Similar NPP4 (PDE4) and PDE4A immunoreactivities were observed mostly to co-localize with PGP 9.5, within nerve fibers running parallel to the smooth muscle bundles, both in the pig (Figs 1A–D and 2A–D) and human (Figs 3A–D and 4A–D) bladder neck. In fact, by using ImageJ software, the quantification of the co-localization between the NPP4, PDE4A and the PGP 9.5, showed values between 75–80% (Figs 1G, 2G, 3G and 4G). No IR was observed in samples processed without the corresponding primary antisera (Figs 1E,F, 2E,F, 3E,F and 4E,F). In western blot analysis, immunoreactive protein bands at 52 kDa and 118 kDa for NPP4 and PDE4A, respectively were detected, thus indicating PDE4 protein expression, essentially of PDE4A isoform, in pig (Figs 1H and 2H) and human (Figs 3H and 4H) bladder neck smooth muscle.

### Effect of CSE and NO synthase blockade and roflumilast on H_2_S generation

**Figure 5:**
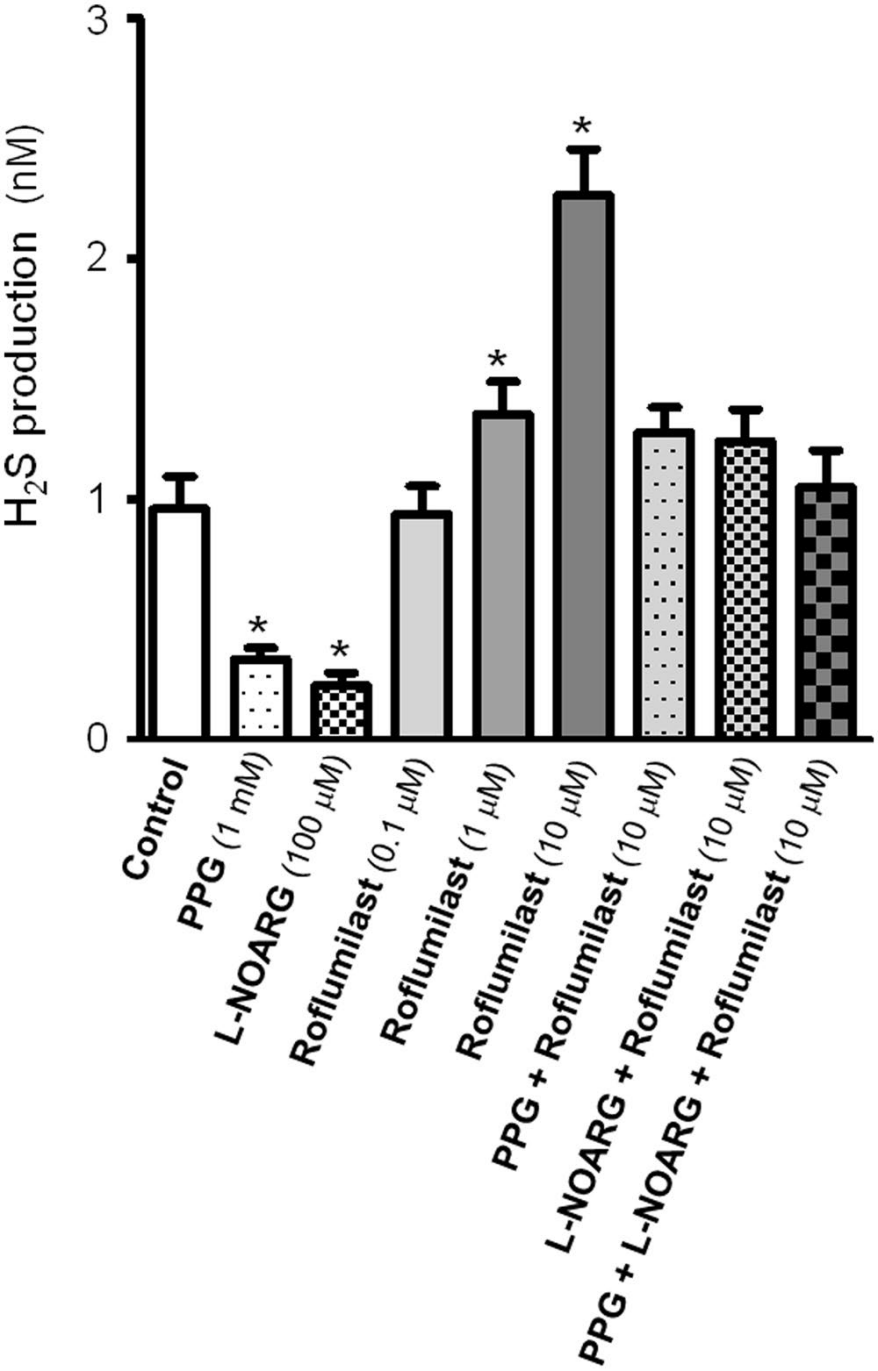
Roflumilast increases endogenous H_2_S production in pig bladder neck samples. Level of H_2_S generated in the absence or presence of DL-propargylglycine (PPG, 1 mM), N^G^-nitro-L-arginine (L-NOARG, 100 µM), inhibitors of CSE and NO synthase, respectively, and the PDE4 inhibitor roflumilast (0.1, 10 and 1 µM), PPG (1 mM) plus roflumilast (10 µM), L-NOARG (100 µM) plus roflumilast (10 µM) and PPG (1 mM) plus L-NOARG (100 µM) plus roflumilast (10 µM). Bars represent mean ± s.e.m. of 8 pigs. ^*^P < 0.05, versus control value (analysis of variance followed by Bonferroni method).

The H_2_S level produced by pig bladder neck samples (0.96 ± 0.13 nM.min-1.g-1) was diminished as consequence of CSE and NO synthase inhibition with, respectively, PPG (1 mM)^24^ and L-NOARG (100 µM)^24^, and recovered by administration of increasing concentrations of roflumilast (0.1–10 µM) (Table 1) (Fig. 5).

**Table 1:** Effect of CSE and NO synthase blockade and roflumilast on endogenous H_2_S production in pig bladder neck samples.

	n	H_2_S level (±nM)
Control	8	0.96 ± 0.13
PPG (1 mM)	8	0.34 ± 0.04^*^
L-NOARG (100 μM)	8	0.23 ± 0.05^*^
Roflumilast (0.1 μM)	8	0.94 ± 0.12
Roflumilast (1 μM)	8	1.36 ± 0.13^*^
Roflumilast (10 μM)	8	2.26 ± 0.19^*^
PPG (1 mM) + Roflumilast (10 μM)	8	1.27 ± 0.11
L-NOARG (100 μM) + Roflumilast (10 μM)	8	1.24 ± 0.13
PPG (1 mM) + L-NOARG (100 μM) + Roflumilast (10 μM)	8	1.05 ± 0.15

Results are expressed as mean ± s.e.m. of *n* experiments. *P < 0.05 versus control (analysis of variance followed by Bonferroni method).

### Functional studies

Strips of pig (n = 36 pigs) and human (n = 4 individuals) bladder neck were equilibrated to a tension of 2.1 ± 0.1 g and 1.9 ± 0.1 g, respectively. PhE (1 μM)^23,24,29^ produced a sustained tone of 2.4 ± 0.1 g (n = 36) and 2.3 ± 0.1 g (n = 4) in pig and human, respectively, strips.

#### Relaxations to roflumilast and tadalafil in pig and human bladder neck

**Figure 6:**
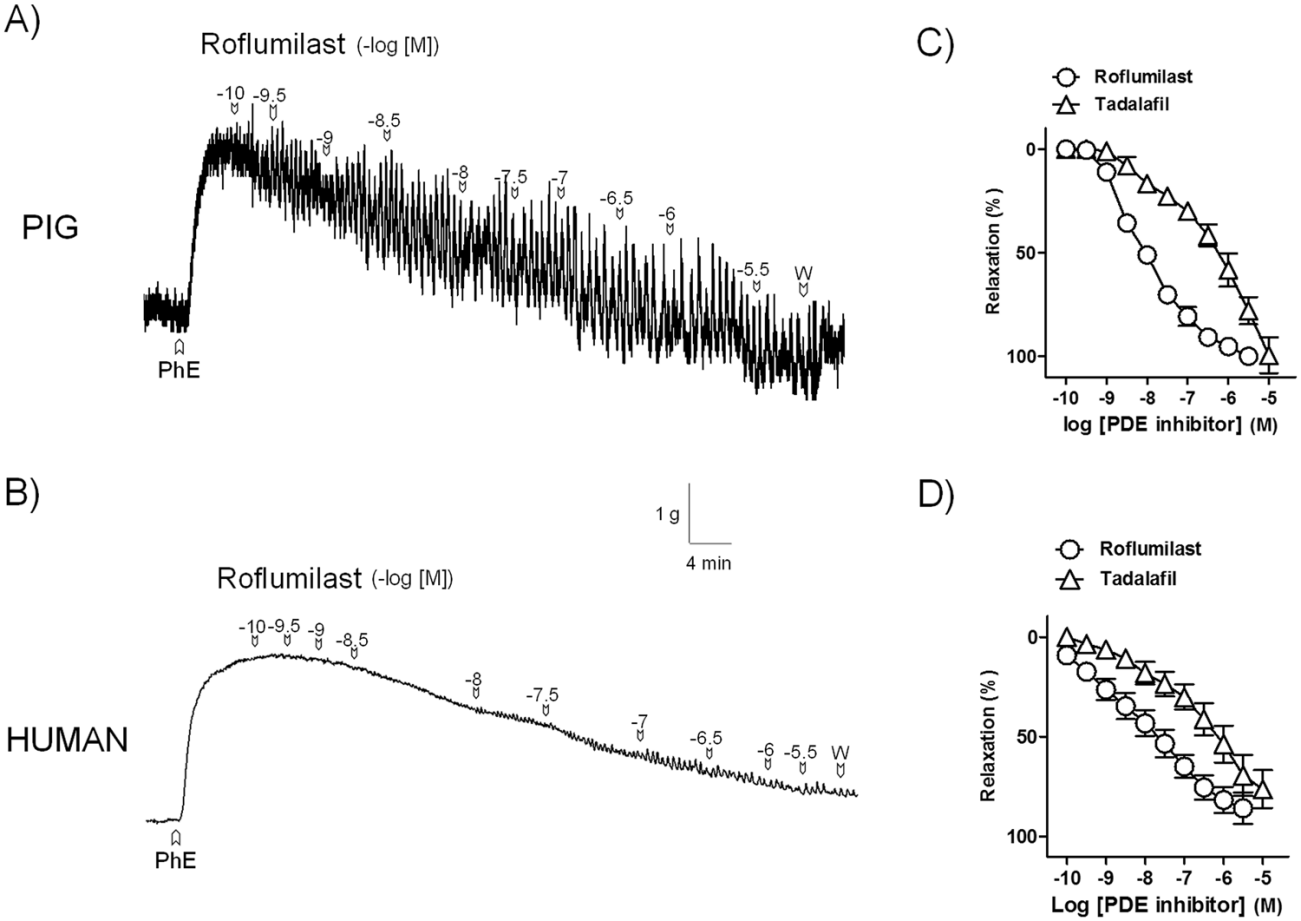
Roflumilast produces a much more potent smooth muscle relaxation versus that induced by tadalafil in pig and human bladder neck. Isometric force recordings showing the relaxations elicited by roflumilast (0.1 nM–3 µM) on 1 µM phenylephrine (PhE)-precontracted urothelium-denuded bladder neck strips of pig (**A**) and human (**B**). Vertical bar shows tension in g and horizontal bar time in min. W: wash. (**C**,**D**) Concentration-response relaxation curves to roflumilast (0.1 nM –3 µM) (open circles) and tadalafil (0.1 nM–10 µM) (open triangles) in pig (n = 8) (**C**) and human (n = 4) (**D**) samples. Results represent mean ± s.e.m.

Roflumilast (0.1 nM–3 μM) and tadalafil (0.1 nM–10 μM) produced relaxations of pig (pD_2_ and Emax values of 8.0 ± 0.1 and 100 ± 2% and 6.0 ± 0.1^*^ and 100 ± 8%, for roflumilast and tadalafil, respectively, ^*^P < 0.05 versus roflumilast value, unpaired *t*–test, n = 8) (Fig. 6A and C) and human (pD_2_ and Emax values of 7.9 ± 0.1 and 85 ± 8% and 6.4 ± 0.1^*^ and 76 ± 9%, for roflumilast and tadalafil, respectively, ^*^P < 0.05 versus roflumilast value, unpaired *t*–test, n = 4) (Fig. 6B and D) bladder neck strips.

#### *Effect of H*_2_*S and NO synthase blockade on roflumilast relaxations*

PPG (1 mM) (pD_2_ and Emax values of 9.1 ± 0.1 and 87 ± 9% and 8.2 ± 0.1^*^ and 76 ± 5%, in the absence or presence, respectively, of PPG, ^*^P < 0.05 versus control value, unpaired *t*–test, n = 5) and L-NOARG (100 µM) (pD_2_ and Emax values of 8.2 ± 0.1 and 83 ± 3% and 7.0 ± 0.1^*^ and 75.0 ± 4%, in the absence or presence, respectively, of L-NOARG, ^*^P < 0.05 versus control, unpaired *t*–test, n = 6), blockers of H_2_S synthase CSE and NO synthase, respectively, reduced roflumilast responses.

#### Effect of threshold roflumilast concentrations on EFS and isoproterenol relaxations

**Figure 7:**
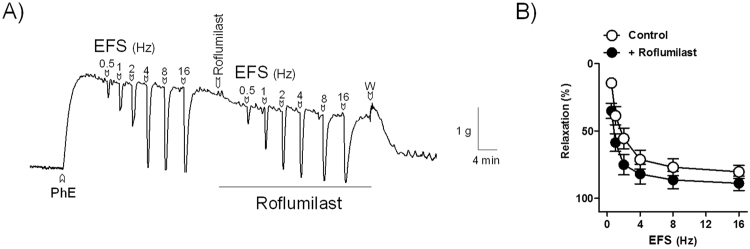
Roflumilast potentiates nerve-mediated relaxation in pig bladder neck. Isometric force recordings showing the relaxations elicited by electrical field stimulation (EFS, 1 ms in duration, 0.5–16 Hz and 20 second trains) in the absence and in the presence of roflumilast (0.6 nM), on 1 μM phenylephrine (PhE)-precontracted urothelium-denuded pig bladder neck strips treated with guanethidine (10 µM) and atropine (1 µM) to block adrenergic neurotransmission and muscarinic receptors, respectively. Vertical bar shows tension in g and horizontal bar time in min. W: wash (**A**). Frequency-response relaxation curves to EFS in the absence (control, open circles) and in the presence (closed circles) of roflumilast (**B**). Results represent mean ± s.e.m. of 9 pigs.

**Figure 8:**
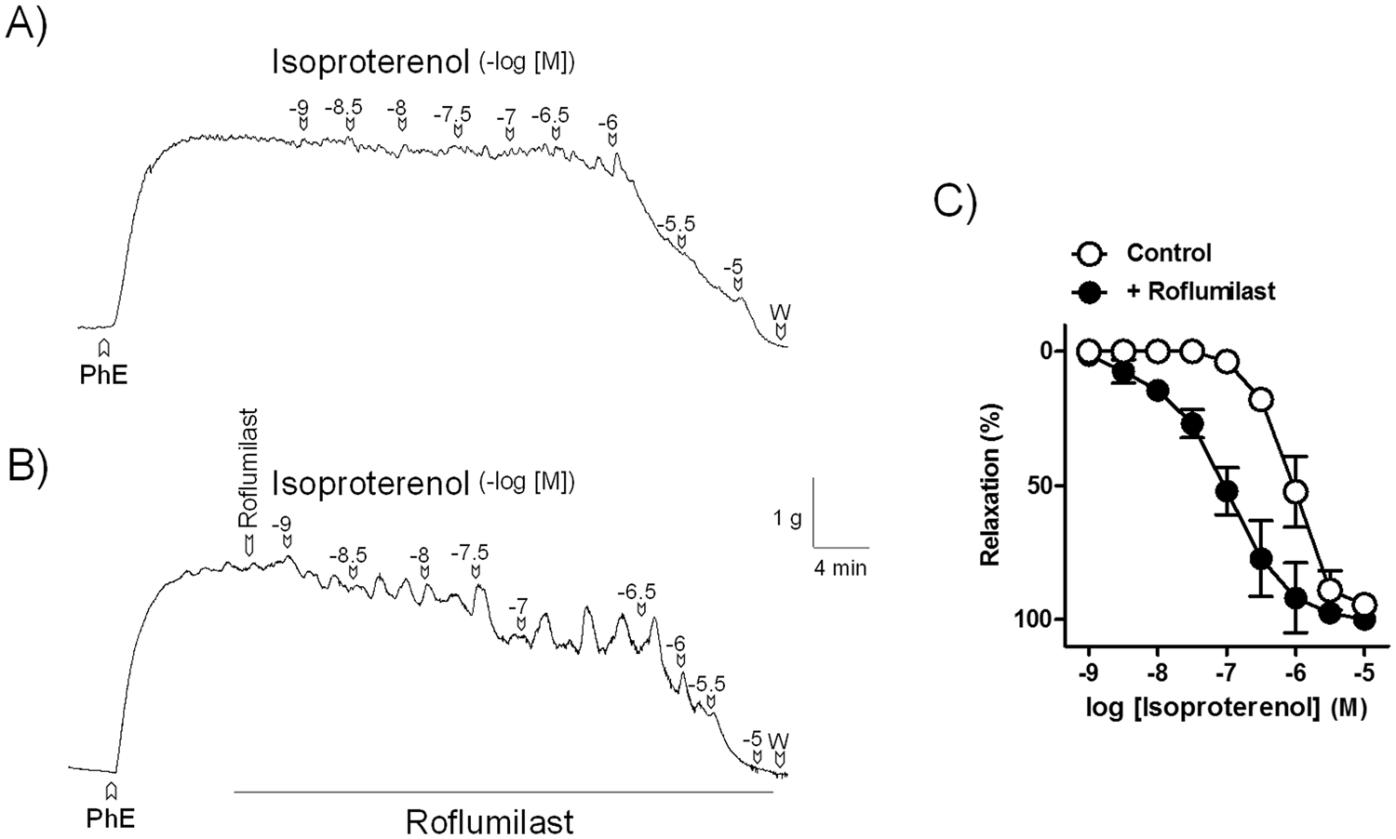
Roflumilast potentiates β-adrenoceptor agonist relaxation in pig bladder neck. Isometric force recordings showing the relaxations elicited by isoproterenol (1 nM–10 µM) in the absence (**A**) and in the presence (**B**) of roflumilast (0.6 nM), on 1 μM phenylephrine (PhE)-precontracted urothelium-denuded pig bladder neck strips. Vertical bar shows tension in g and horizontal bar time in min. W: wash (**A**). Concentration-response relaxation curves to isoproterenol in the absence (control, open circles) and in the presence (closed circles) of roflumilast (**C**). Results represent mean ± s.e.m. of 8 pigs.

On PhE-induced contraction of pig bladder neck strips, under NANC conditions, EFS induced frequency-dependent relaxations, which were increased by the pre-treatment with threshold (0.6 nM) roflumilast concentrations (relaxations evoked by EFS at 0.5, 1 and 2 Hz were of 14 ± 3%, 39 ± 7% 56 ± 8% and 35 ± 6%^*^, 59 ± 7%^*^ and 75 ± 8%^*^ in the absence or presence of roflumilast, respectively, ^*^P < 0.05 versus control, paired *t*–test, n = 9) (Fig. 7). In addition, roflumilast also potentiated the isoproterenol relaxations (pD_2_ and Emax values of 6.1 ± 0.1 and 95 ± 1% and 7.0 ± 0.1^*^ and 100 ± 0%, in the absence or presence of roflumilast, respectively, ^*^P < 0.05 versus control value, unpaired *t*–test, n = 8) (Fig. 8).

## Discussion

The main finding of the present study is that neuronal PDE4, essentially PDE4A isoform, plays a crucial role in regulating contractility of the pig and human bladder outflow region. In fact, PDE4 inhibition and subsequent increased neuronal [cAMP] produces a potent smooth muscle relaxation due to its facilitatory action on nerve-mediated response. The above conclusion is maintained by the following data: (1) NPP4 and PDE4A are mainly detected within nerve fibers distributed along the smooth muscle layer of the pig and human bladder neck, where roflumilast exerts a much more potent relaxation compared to that induced by the PDE5 inhibitor tadalafil. (2) In pig bladder neck membranes, endogenous H_2_S production was reduced by H_2_S and NO synthesis enzyme blockade and increased by roflumilast. (3) Roflumilast relaxation was also diminished by H_2_S and NO synthesis blockade. (4) EFS- and isoproterenol-induced relaxations were enhanced in the presence of roflumilast threshold concentrations.

Several PDE isoenzymes, such as PDE1A, PDE4A, PDE4B and PDE5A have been identified in the human urethra and prostatic smooth muscle, where selective inhibitors of PDE4 and PDE5 cause relaxation, thus indicating that, in addition to the NO/cGMP/PKG pathway, the cAMP/PKA-dependent signaling might be a valuable target to treat symptoms of LUTS/BPE^34–36^. In the present study, in the pig and human bladder neck samples, PDE4 (NPP4) and PDE4A expression were consistently detected. In fact, western blot analysis in the pig and human bladder neck samples showed a consistent PDE4 (NPP4) and PDE4A protein expression. Moreover, double-labeling immunofluorescence assays revealed IR for NPP4 and PDE4A as nerve fibers running alongside the smooth muscle layer. These results agree with those obtained in the human seminal vesicles, where immunosignals specific for PDE4 co-localized with vasoactive intestinal peptide were found in nerve fibers and nerve endings^37^. In our study, the presence of NPP4 and PDE4A within nerve fibers also suggested a modulatory role for PDE4 in the inhibitory efferent neurotransmission of the bladder neck. Selective PDE5 inhibitors change the endogenous production and/or effect of neurotransmitters in the lower urinary tract. In fact, in human detrusor, sildenafil causes smooth muscle relaxation and increases the H_2_S generation, thus indicating that PDE5 inhibitor relaxation on the human bladder involves the H_2_S signaling pathway^38^. Moreover, tadalafil enhances the inhibitory effect of the α_1A_-adrenergic receptor antagonist tamsulosin on EFS-elicited neurogenic contraction of human bladder neck^39^. In the pig bladder neck, NO and H_2_S play a pivotal role in the NANC inhibitory neurotransmission. NO, whose release from intramural nerves is modulated by pre-junctional α_2_-adrenoceptor and voltage-gated K^+^ channel activation, relaxes smooth muscle through a mechanism dependent on the activation of guanylyl cyclase, in which post-junctional K^+^ channels do not seem to be involved^20,21^. In addition, H_2_S, synthesized by CSE and released from nerves, produces bladder neck smooth muscle relaxation through opening of ATP-dependent K^+^ channels and by intracellular Ca^2+^ desensitization-dependent mechanisms. H_2_S also promotes the release of neuropeptides and cyclooxygenase-1 pathway-derived prostanoids from capsaicin sensitive primary afferents, via transient receptor potential A1, transient receptor potential vanilloid 1 and/or related ion channel activation^23,24^. In the present study, in the pig bladder neck, H_2_S synthesis enzyme CSE and NO synthase blockade diminished the endogenous H_2_S production, thus indicating that H_2_S is generated through activation of CSE, also involving a NO synthetic pathway. H_2_S production was, nervetheless, completely restored and significantly increased, in a dose-dependent manner, above control sample levels, as a consequence of stimulation with increasing roflumilast concentrations, thus indicating that PDE4 isoenzyme, present in the nerves, would be involved in regulating production and/or release of gaseous inhibitory neurotransmitter/s. Since PDE4 inhibition enhanced H_2_S generation, these results suggest a facilitatory role for cAMP on neurotransmitter release in the bladder neck.

The fact that roflumilast relaxation was much more potent than that induced by tadalafil agrees with that described in human urethra, where PDE4 inhibitors exhibit a higher rank order of efficacy versus that shown by the PDE5 inhibitors sildenafil, vardenafil and tadalafil^35^. Since NO^20,21^ and H_2_S^23,24^ are the major NANC inhibitory neurotransmitters in pig bladder neck, the samples in this study were treated with NO synthase and H_2_S synthase CSE inhibitors, which reduced the roflumilast response, thus suggesting that PDE4 inhibition and the subsequent increase in neuronal cAMP levels may favour the release of NO and H_2_S. This fact, together with the potentiation of EFS-elicited neurogenic relaxation observed in presence of a threshold roflumilast concentration and the marked expression of PDE4, especially PDE4A, within nerve fibers, suggests that pig bladder neck roflumilast relaxation is produced via neuronal H_2_S- and NO-dependent signaling pathways. These results agree with those described in human detrusor, where the L-cysteine/H_2_S pathway is involved in the relaxation produced by PDE5 inhibitors. In fact, not only stable analogs of cGMP but also cAMP elevate the H_2_S levels in the human bladder, thus supporting the role of cAMP/PKA signaling pathway in the H_2_S-mediated relaxation^38^.

In airways smooth muscle, an interaction between PDE4 inhibitors and beta-adrenoceptor agonists has been documented. In fact, PDE4 inhibitors, such as rolipram and quercetin, cause a potent smooth muscle relaxation of human bronchus and mice trachea, respectively, and potentiate β-adrenoceptor stimulation relaxation^40,41^. In the current study, a roflumilast threshold concentration evoked a leftward displacement of the relaxation concentration-response curve to the β-adrenoceptor agonist isoproterenol, thus suggesting that PDE4 inhibitors, in addition to cause a powerful smooth muscle relaxation *per se*, enhance the relaxant effect produced via β-adrenoceptor stimulation in the bladder outlet region.

Selective PDE4 inhibitors also attenuate the development of cyclophosphamide-induced cystitis improving the bladder symptoms, as voiding behaviour and histological damage by suppressing cytokine production and inducible NO synthase induction, and causing a significant decrease on the amplitude and frequency of spontaneous contractions without changing cystometric parameters, thus suggesting that PDE4 inhibitors might be an attractive strategy for the treatment of chemically-induced bladder overactivity^42–45^. Therefore, PDE4 inhibitory activity might provide, in addition to relaxant action, an anti-inflammatory therapy for bladder urine outflow region. The fact that roflumilast, an orally active anti-inflammatory drug used as an add-on treatment to long-acting bronchodilators has been approved for the treatment of severe COPD^30–33^, suggests the possible usefulness of roflumilast and/or its active metabolite N-oxide, alone or in combination with the PDE5 inhibitors or α_1A_-adrenoceptor antagonists for smooth muscle relaxation of lower urinary tract outflow region in patients suffering LUTS/BPE.

In conclusion, current results suggest that PDE4, predominantly PDE4A, is mostly expressed within nerve fibers of the pig and human bladder neck, where roflumilast promotes a much more powerful smooth muscle relaxation in comparison with that produced by tadalafil. In pig, the fact that roflumilast increases endogenous H_2_S production and EFS relaxation suggests that PDE4 inhibition and subsequent increased neuronal [cAMP] facilitates NO- and H_2_S-mediated bladder neck inhibitory neurotransmission. To our knowledge this is the first time that it is suggested a modulatory role for neuronal PDE4 in the gaseous inhibitory transmission.

## Methods

### Tissue

Urinary bladders from adult male pigs were provided from the Matadero Madrid Norte slaughterhouse. Human bladder samples were taken from 4 men. One of them was 53 years old and died as consequence of a cerebrovascular injury, and another 43 years old who died in a traffic accident. Human samples were also obtained from two other patients, 64- and 56-year old, who had undergone cystectomy for bladder cancer. All methods were carried out in accordance with relevant guidelines and regulations. The study protocol with human samples was approved by the Ethics Committees from Hospital Universitario Puerta de Hierro-Majadahonda and Hospital Universitario Ramón y Cajal de Madrid (Madrid, Spain), with informed consent obtained from all subjects or families of the organ donors. Bladders were maintained in physiological saline solution at 4 °C.

### Western Blot

Proteins extracted from pig and human bladder neck (50 μg of protein) were separated by 10% polyacrylamide gel (SDS-PAGE), after which the transfer membranes were exposed to specific antibodies against NNP4 and PDE4A (Santa Cruz Biotechnology Inc. Heidelberg, Germany) at a 1:50 dilution. Mouse anti-β-actin antibody was used as a loading control (Santa Cruz Biotechnology Inc. Heidelberg, Germany). Membranes were incubated with the secondary antibody (1:5000 dilution); the blots were detected with an ECL solution (ECL Select-kit, GE Healthcare), using an Image-Quant LAS 500 system chemiluminescence imaging system (GE Healthcare).

### Double-labeling immunofluorescence assays

Pig and human bladder neck segments were placed in an solution of 4% paraformaldehyde in phosphate buffer (PB; pH 7.4), and subsequently placed in 30% sucrose in PB for cryoprotection. The tissue was sliced into transversal sections 5 μm thickness and preincubated in 10% normal donkey serum in PB containing 0.3% Triton-X-100, for 2–3 h. Followed the sections were incubated with either goat anti-PDE4 (anti-NPP4 C-15, Santa Cruz Biotechnology Inc. Heidelberg, Germany) at a 1:50 dilution plus a mouse anti-protein gene product 9.5 (anti-PGP 9.5), diluted 1:100 and mouse anti PDE4A (anti-PDE4A H-7, Santa Cruz Biotechnology Inc. Heidelberg, Germany) at a 1:50 dilution plus a rabbit anti-protein gene product 9.5 (anti-PGP 9.5) diluted 1:50 during 48 h at 4 °C. After rinsing the sections with PB, fluorochrome-labeled secondary antibodies were applied for 3 h at room temperature (Alexa Fluor 594 donkey-anti-rabbit, Alexa Fluor 594 goat-anti-rabbit and Alexa Fluor 488 goat-anti mouse, 1:200 dilution) (Abcam). The slides were mounted with DAPI (Invitrogen), which stains all cell nuclei.

On the original raw images, the intensity of immunostainining was measured using ImageJ free software (National Institutes of Health, USA). All microphotographs were obtained in the same conditions.

### Endogenous H_2_S measurement

The method used for measuring endogenous production and release of H_2_S was previously described^46^. Briefly, tissues were homogenized (1:10 w/v) in ice-cold 50 mM potassium phosphate, pH 6.8. The incubation solution (1 ml in each incubation chamber) contained of 10 mM L-cysteine, 2 mM pyridoxal 5′-phosphate, 100 mM potassium phosphate buffer, pH 7.4, and 50 mg of tissue homogenate. DL-propargylglycine (PPG, 1 mM), N^G^-nitro-L-arginine (L-NOARG, 100 μM) or roflumilast (0.1, 1 and 10 μM) were added to the incubation solution in order to examine the role of an irreversible CSE inhibitor, a NO synthase inhibitor, and the selective PDE4 inhibitor, respectively. The reaction was initiated by transfer of the tubes from ice to a shaking water bath at 37 °C. After incubation for 30 min, 1% zinc acetate was injected to trap-generated H2S, and, then the reaction was stopped by applying 500% (w/v) trichloroacetic acid to denature the protein. Subsequently, N,N-dimethyl-p-phenylenediamine sulfate (20 mM; 0.5 ml) in 7.2 M HCl was added, immediately followed by FeCl_3_ (30 mM; 0.4 ml) in 1.2 M HCl. The absorbance of the resulting solution at 670 nm was measured 20 min later by spectrophotometry. The H2S concentration was calculated against a calibration curve of NaHS standard. Results are expressed as nanomoles of H_2_S formed per mg of soluble protein per 20 min. Protein was determined with the Lowry assay (DC Protein Assay Kit, Bio-Rad, Madrid, Spain).

### Isometric force recording

Pig and human bladders were kept in chilled physiological saline solution (PSS) at 4 °C. Urothelium and suburothelium were removed by microdissection under the microscope. The absence of these layers was confirmed by histological study. Bladder neck strips (4–6 mm long and 2–3 mm wide) were suspended horizontally in 5 ml organ baths containing PSS at 37 °C and continuously gassed with 95% O_2_ and 5% CO_2_ obtaining a final pH of 7.4. One end of the preparation was connected to an isometric force transducer (Grass FT03C) and the other one to a micrometer screw, with the signal continuously recorded on a polygraph (Graphtec Multicorder MC6621). The strips were allowed to equilibrate, under a passive tension of 2.0 g, for about 60 min in PSS and washed with (37 °C) PSS at 20 min intervals. The contractile capacity of the preparations was tested by stimulating them with 124 mM potassium-rich PSS (124 mM KPSS). In strips precontracted with 1 μM phenylephrine (PhE) concentration-response relaxation curves to roflumilast, tadalafil and isoproterenol, PDE4 and PDE5 inhibitors, and β-adrenergic receptor agonist, respectively, were constructed. Due to that relaxation curves to roflumilast were not reproducible, preparations from the same animal were run in parallel, using one of them as control, and the other one to assess the specific treatment for 30 min.

In electrical field stimulation (EFS) assays, pig bladder neck strips were pre-treated with guanethidine (10 μM) and atropine (1 μM) in order to block noradrenergic neurotransmission and muscarinic receptors, respectively, for 1 h, replacing the solution every 20 min, and these drugs were present throughout the experiment. Under these conditions and after 1 μM PhE-induced tone, relaxations to EFS in the absence or presence of threshold roflumilast concentrations were carried out in the same preparation. EFS was carried out using a stimulator (CS20, Cibertec, Barcelona, Spain), connected to two platinum electrodes placed on each side of the strip in parallel to its longitudinal axis, by delivering rectangular pulses (1 ms duration, 0.5–16 Hz, 20 s trains, with constant current output adjusted to 75 mA), at 4 min intervals. Control EFS curves run parallel.

### Drugs and solutions

The drugs used were: Isoproterenol, N^G^-nitro-L-arginine (L-NOARG), phenylephrine (PhE), DL-propargylglycine (PPG), roflumilast and tadalafil, all from Sigma (USA). PPG and roflumilast were dissolved in dimethylsulphoxide. Tadalafil was dissolved in acetonitrile. The other compounds were dissolved in double-distilled water. Preliminary experiments revealed no effects of the solvents used on bladder neck samples contractility.

PSS composition (in mM)was: NaCl 119, KCl 4.6, MgCl_2_ 1.2, NaHCO_3_ 24.9, glucose 11, CaCl_2_ 1.5, KH_2_PO_4_ 1.2, ethylenediamine tetraacetic acid (EDTA) 0.027. PSS was maintained at 37 °C and gassed with 95% O_2_ and 5% CO_2_, thus obtaining a final pH of 7.4 in the organ bath. To prepare K^+^-enriched PSS (KPSS), the NaCl in PSS was replaced with KCl on an equimolar basis.

### Calculations and statistics

Sensitivity to roflumilast, tadalafil and isoproterenol is expressed in terms of pD_2_, where pD_2_ = −log EC_50_ and EC_50_ is the agonist concentration needed to produce half-maximal response. pD_2_ was estimated by computerized non-linear regression analysis (GraphPad Prism, USA). Relaxations are shown as a percent of the pre-contraction induced by PhE. Results are expressed as the mean ± s.e.m. of *n* (number of pigs or persons). Differences were analyzed using one-way analysis of variance (ANOVA) or paired or unpaired Student’s *t*-test when appropriate. Probability levels less than 5% were considered significant (*P* < 0.05). The calculations were carried out by using a standard software (GraphPad Prism 6.01, San Diego, CA).

## Electronic supplementary material

Dataset 1

## References

[1] Matsumoto T, Kobayashi T, Kamata K (2003). Phosphodiesterases in the vascular system. J. Smooth Muscle Res..

[2] Wheeler MA, Ayyagari RR, Wheeler GL, Weiss RM (2005). Regulation of cyclic nucleotides in the urinary tract. J. Smooth Muscle Res..

[3] Petkov GV, Nelson MT (2005). Differential regulation of Ca^2+^-activated K^+^ channels by beta-adrenoceptors in guinea pig urinary bladder smooth muscle. Am J Physiol Cell Physiol.

[4] Oger S (2007). Relaxation of phasic contractile activity of human detrusor strips by cyclic nucleotide phosphodiesterase type 4 inhibition. Eur. Urol..

[5] Nishiguchi J (2007). Suppression of detrusor overactivity in rats with bladder outlet obstruction by a type 4 phosphodiesterase inhibitor. B.J.U. Int..

[6] Brown SM (2008). Beta-adrenergic relaxation of mouse urinary bladder smooth muscle in the absence of large-conductance Ca^2+^-activated K^+^ channel. Am. J. Physiol. Renal Physiol..

[7] Hristov KL (2008). Stimulation of beta3-adrenoceptors relaxes rat urinary bladder smooth muscle via activation of the large-conductance Ca^2+^-activated K^+^ channels. Am. J. Physiol. Cell. Physiol..

[8] Ückert S (2009). Significance of phosphodiesterase isoenzymes in the control of human detrusor smooth muscle function. An immunohistochemical and functional study. Urologe A.

[9] Andersson KE (2007). LUTS treatment: future treatment options. Neurourol. Urodyn..

[10] Andersson KE, Ückert S, Stief C, Hedlund P (2007). Phosphodiesterases (PDEs) and PDE inhibitors for treatment of LUTS. Neurourol. Urodyn..

[11] Kaiho Y (2008). The effects of a type 4 phosphodiesterase inhibitor and the muscarinic cholinergic antagonist tolterodine tartrate on detrusor overactivity in female rats with bladder outlet obstruction. B.J.U. Int..

[12] Andersson KE (2011). Tadalafil for the treatment of lower urinary tract symptoms secondary to benign prostatic hyperplasia: pathophysiology and mechanism(s) of action. Neurourol. Urodyn..

[13] Gacci M (2012). A randomized, placebo-controlled study to assess safety and efficacy of vardenafil 10 mg and tamsulosin 0.4 mg vs. tamsulosin 0.4 mg alone in the treatment of lower urinary tract symptoms secondary to benign prostatic hyperplasia. J. Sex. Med..

[14] Giuliano F (2013). Tadalafil once daily improves ejaculatory function, erectile function, and sexual satisfaction in men with lower urinary tract symptoms suggestive of benign prostatic hyperplasia and erectile dysfunction: results from a randomized, placebo- and tamsulosin-controlled, 12-week double-blind study. J. Sex. Med..

[15] Giuliano F (2013). The mechanism of action of phosphodiesterase type 5 inhibitors in the treatment of lower urinary tract symptoms related to benign prostatic hyperplasia. Eur. Urol..

[16] Yamaguchi O (2013). Latest treatment for lower urinary tract dysfunction: therapeutic agents and mechanism of action. Int. J. Urol..

[17] Rahnama’I MS, Ückert S, Hohnen R, van Koeveringe GA (2013). The role of phosphodiesterases in bladder pathophysiology. Nat. Rev. Urol..

[18] Dmochowski R (2013). Urodynamic effects of once daily tadalafil in men with lower urinary tract symptoms secondary to clinical benign prostatic hyperplasia: a randomized, placebo controlled 12-week clinical trial. J. Urol..

[19] Gacci M (2016). Latest Evidence on the Use of Phosphodiesterase Type 5 Inhibitors for the Treatment of Lower Urinary Tract Symptoms Secondary to Benign Prostatic Hyperplasia. Eur. Urol..

[20] Hernández M (2008). Role of neuronal voltage-gated K^+^ channels in the modulation of the nitrergic neurotransmission of the pig urinary bladder neck. Br. J. Pharmacol..

[21] Hernández M (2007). Pre-junctional alpha2-adrenoceptors modulation of the nitrergic transmission in the pig urinary bladder neck. Neurourol. Urodyn..

[22] Bustamante S (2010). Functional evidence of nitrergic neurotransmission in the human urinary bladder neck. Neurosci. Lett..

[23] Fernandes VS (2013). Hydrogen sulfide-mediated inhibitory neurotransmission to the pig bladder neck: Role of KATP channels, sensory nerves and calcium signaling. J. Urol..

[24] Fernandes VS (2013). Endogenous hydrogen sulfide has a powerful role in inhibitory neurotransmission to the pig bladder neck. J. Urol..

[25] Hernández M, Knight GE, Wildman SS, Burnstock G (2009). Role of ATP and related purines in inhibitory neurotransmission to the pig urinary bladder neck. Br. J. Pharmacol..

[26] Recio P (2009). 5-hydroxytryptamine induced relaxation in the pig urinary bladder neck. Br. J. Pharmacol..

[27] Hernández M (2006). Neuronal and smooth muscle receptors involved in the PACAP- and VIP-induced relaxations of the pig urinary bladder neck. Br. J. Pharmacol..

[28] Hernández M (2006). PACAP 38 is involved in the non adrenergic non cholinergic inhibitory neurotransmission in the pig urinary bladder neck. Neurourol. Urodyn..

[29] Ribeiro AS (2014). Powerful relaxation of phosphodiesterase type 4 inhibitor rolipram in the pig and human bladder neck. J. Sex. Med..

[30] Wollin L, Bundschuh DS, Wohlsen A, Marx D, Beume R (2006). Inhibition of airway hyperresponsiveness and pulmonary inflammation by roflumilast and other PDE4 inhibitors. Pulm. Pharmacol. Ther..

[31] Venkatasamy R, Spina D (2016). Novel relaxant effects of RPL554 on guinea pig tracheal smooth muscle contractility. Br. J. Pharmacol..

[32] Bateman ED, Goehring UM, Richard F, Watz H (2016). Roflumilast combined with montelukast versus montelukast alone as add-on treatment in patients with moderate-to-severe asthma. J. Allergy Clin. Immunol..

[33] Yuan L, Dai X, Yang M, Cai Q, Shao N (2016). Potential treatment benefits and safety of roflumilast in COPD: a systematic review and meta-analysis. Int. J. Chron. Obstruct. Pulmon. Dis..

[34] Waldkirch E (2010). Expression of cAMP-dependent protein kinase isoforms in the human prostate: functional significance and relation to PDE4. Urology.

[35] Kedia GT (2014). Phosphodiesterase isoenzymes in the human urethra: a molecular biology and functional study. Eur. J. Pharmacol..

[36] Kedia GT (2015). Expression and distribution of phosphodiesterase isoenzymes in the human male urethra. Urology.

[37] Ückert S (2011). Expression and distribution of phosphodiesterase isoenzymes in the human seminal vesicles. J. Sex. Med..

[38] Fusco F (2012). Sildenafil effect on the human bladder involves the L-cysteine/hydrogen sulfide pathway: a novel mechanism of action of phosphodiesterase type 5 inhibitors. Eur. Urol..

[39] Angulo J (2012). Tadalafil enhances the inhibitory effects of tamsulosin on neurogenic contractions of human prostate and bladder neck. J. Sex. Med..

[40] Qian Y, Naline E, Karlsson JA, Raeburn D, Advenier C (1993). Effects of rolipram and siguazodan on the human isolated bronchus and their interaction with isoprenaline and sodium nitroprusside. Br. J. Pharmacol..

[41] Townsend EA, Emala CW (2013). Quercetin acutely relaxes airway smooth muscle and potentiates β-agonist-induced relaxation via dual phosphodiesterase inhibition of PLCβ and PDE4. Am. J. Physiol. Lung Cell. Mol. Physiol..

[42] Büyüknacar HS, Kumcu EK, Göçmen C, Onder S (2008). Effect of phosphodiesterase type 4 inhibitor rolipram on cyclophosphamide-induced cystitis in rats. Eur. J. Pharmacol..

[43] Kitta T, Tanaka H, Mitsui T, Moriya K, Nonomura K (2008). Type 4 phosphodiesterase inhibitor suppresses experimental bladder inflammation. B.J.U. Int..

[44] Sakura M (2009). Rolipram, a specific type-4 phosphodiesterase inhibitor, inhibits cyclophosphamide-induced haemorrhagic cystitis in rats. B.J.U. Int..

[45] Li K (2013). Eviprostat activates cAMP signaling pathway and suppresses bladder smooth muscle cell proliferation. Int. J. Mol. Sci..

[46] Li L (2005). Hydrogen sulfide is a novel mediator of lipopolysaccharide-induced inflammation in the mouse. FASEB J..

