# Supplementary material for: Phosphodiesterase type 4 inhibition enhances nitric oxide- and hydrogen sulfide-mediated bladder neck inhibitory neurotransmission
Source: Sci Rep. 2018 Mar 16;8:4711. doi: 10.1038/s41598-018-22934-1 (PMC5856743; doi:10.1038/s41598-018-22934-1)

**Phosphodiesterase type 4 inhibition enhances nitric oxide- and hydrogen sulfide-mediated bladder neck inhibitory neurotransmission.**

Ángel Agis-Torres, Paz Recio, María Elvira López-Oliva, María Pilar Martínez, María Victoria Barahona, Sara Benedito, Salvador Bustamante, Miguel Ángel Jiménez-Cidre, Albino García-Sacristán, Dolores Prieto, Vítor S Fernandes, Medardo Hernández.


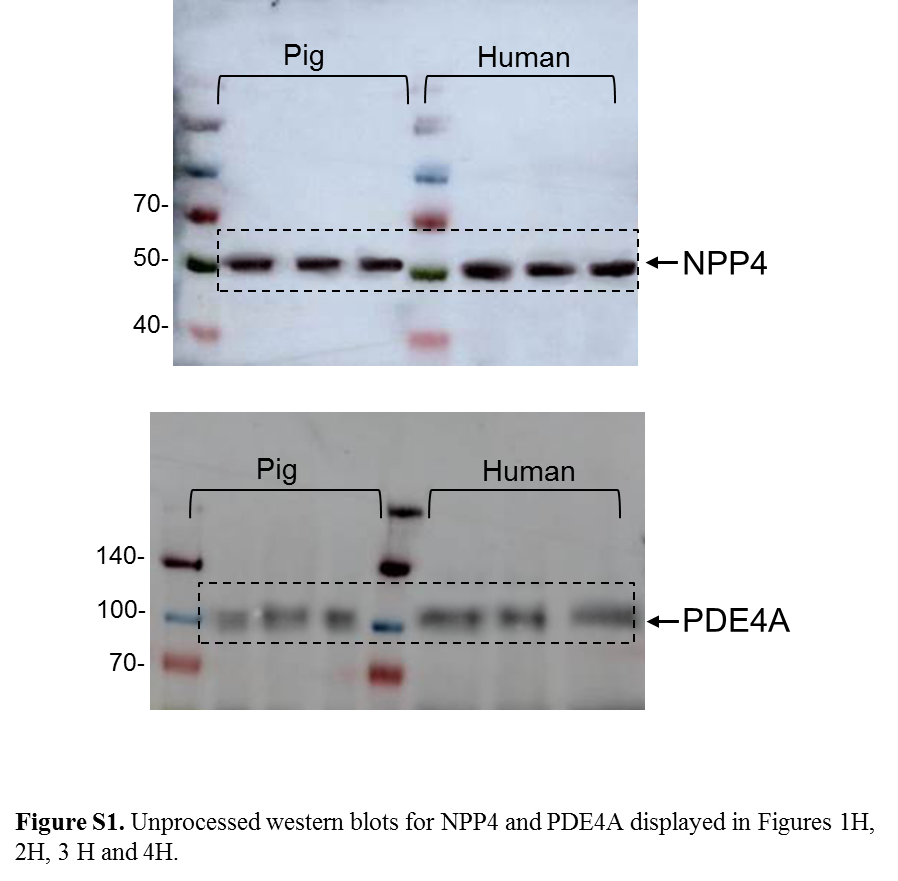

Supplement: Supplementary file 1 — Dataset 1 [file 41598_2018_22934_MOESM1_ESM.docx]
